# Supplementary material for: Causal Relationship between Aging and Anorexia Nervosa: A White-Matter-Microstructure-Mediated Mendelian Randomization Analysis
Source: Biomedicines. 2024 Aug 16;12(8):1874. doi: 10.3390/biomedicines12081874 (PMC11351342; doi:10.3390/biomedicines12081874)
Supplement: Supplementary file 1 [file biomedicines-12-01874-s001.zip › Supplementary Figure/Figure S4/Singe SNP plot of MR analysis for TL to ubm-b-1832.pdf]

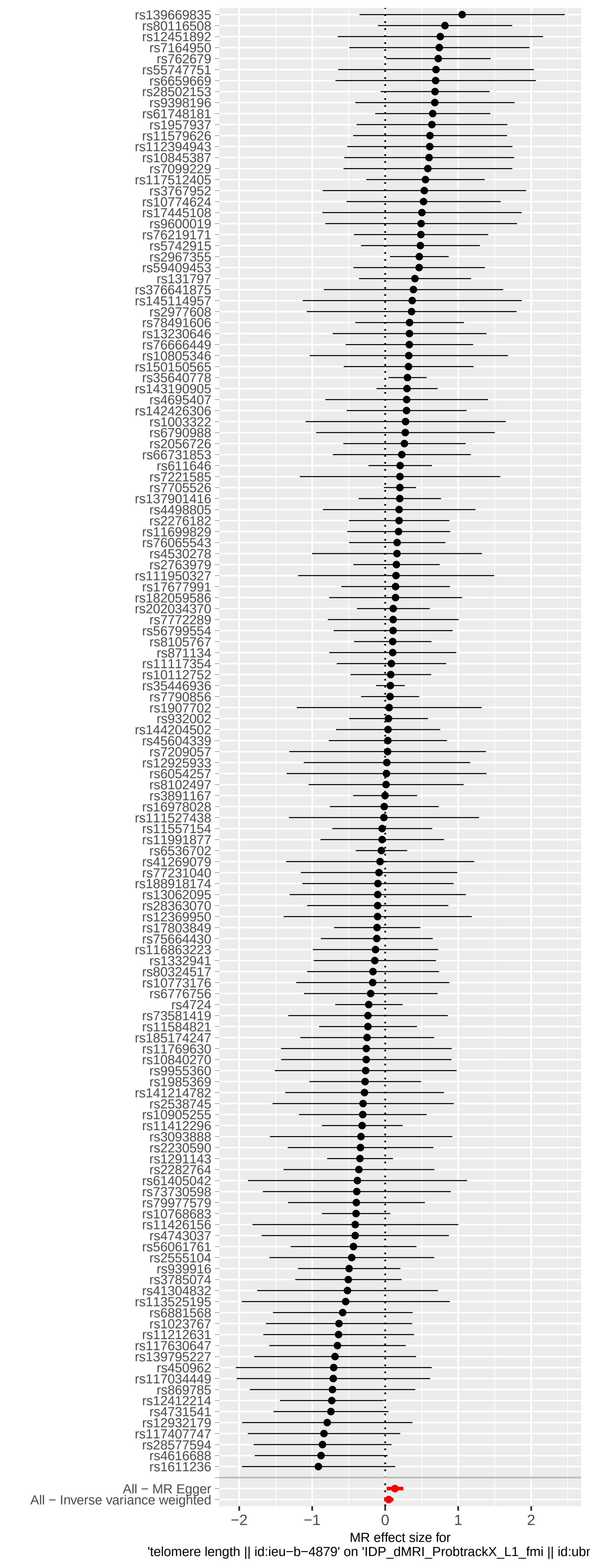
rs139669835
rs80116508
rs12451892
rs7164950
rs762679
rs55747751
rs6659669
rs28502153
rs9398196
rs61748181
rs1957937
rs11579626
rs112394943
rs10845387
rs7099229
rs117512405
rs3767952
rs10774624
rs17445108
rs9600019
rs76219171
rs5742915
rs2967355
rs59409453
rs131797
rs376641875
rs145114957
rs2977608
rs78491606
rs13230646
rs76666449
rs10805346
rs150150565
rs35640778
rs143190905
rs4695407
rs142426306
rs1003322
rs6790988
rs2056726
rs66731853
rs611646
rs7221585
rs7705526
rs137901416
rs4498805
rs2276182
rs11699829
rs76065543
rs4530278
rs2763979
rs111950327
rs17677991
rs182059586
rs202034370
rs7772289
rs56799554
rs8105767
rs871134
rs11117354
rs10112752
rs35446936
rs7790856
rs1907702
rs932002
rs144204502
rs45604339
rs7209057
rs12925933
rs6054257
rs8102497
rs3891167
rs16978028
rs111527438
rs11557154
rs11991877
rs6536702
rs41269079
rs77231040
rs188918174
rs13062095
rs28363070
rs12369950
rs17803849
rs75664430
rs116863223
rs1332941
rs80324517
rs10773176
rs6776756
rs4724
rs73581419
rs11584821
rs185174247
rs11769630
rs10840270
rs9955360
rs1985369
rs141214782
rs2538745
rs10905255
rs11412296
rs3093888
rs2230590
rs1291143
rs2282764
rs61405042
rs73730598
rs79977579
rs10768683
rs11426156
rs4743037
rs56061761
rs2555104
rs939916
rs3785074
rs41304832
rs113525195
rs6881568
rs1023767
rs11212631
rs117630647
rs139795227
rs450962
rs117034449
rs869785
rs12412214
rs4731541
rs12932179
rs117407747
rs28577594
rs4616688
rs1611236
All − MR Egger
All − Inverse variance weighted
−2
−1
0
1
2
MR effect size for
'telomere length || id:ieu−b−4879' on 'IDP_dMRI_ProbtrackX_L1_fmi || id:ubr
